# Fabrication of Bilayer Magnetically Actuated L-Shaped Microrobot Based on Chitosan via Photolithography

**DOI:** 10.3390/polym14245509

**Published:** 2022-12-15

**Authors:** Haoying Wang, Xiaoxia Song, Junfeng Xiong, U Kei Cheang

**Affiliations:** 1 Department of Mechanical and Energy Engineering, Southern University of Science and Technology, Shenzhen 518055, China; 2 Shenzhen Key Laboratory of Biomimetic Robotics and Intelligent Systems, Southern University of Science and Technology, Shenzhen 518055, China; 3 Guangdong Provincial Key Laboratory of Human-Augmentation and Rehabilitation Robotics in Universities, Southern University of Science and Technology, Shenzhen 518055, China

**Keywords:** chitosan, microrobot, photolithography, natural polymer, magnetic actuation

## Abstract

Magnetically actuated microrobots showed increasing potential in various fields, especially in the biomedical area, such as invasive surgery, targeted cargo delivery, and treatment. However, it remains a challenge to incorporate biocompatible natural polymers that are favorable for practical biomedical applications. In this work, bilayer magnetic microrobots with an achiral planar design were fabricated using a biocompatible natural polymer and Fe_3_O_4_ nanoparticles through the photolithography by applying the layer-by-layer method. The microrobots consisted of a magnetic bottom layer and a photo-crosslinked chitosan top layer. The SEM results showed that the microrobot processed the L-shaped planar structure with the average width, length, and thickness of 99.18 ± 5.11 μm, 189.56 ± 11.37 μm, and 23.56 ± 4.08 μm, respectively. Moreover, microrobots actuated using a three-dimensional (3D) Helmholtz coil system was characterized and reached up to an average maximum velocity of 325.30 μm/s and a step-out frequency of 14 Hz. Furthermore, the microrobots exhibited excellent cell biocompatibility towards L929 cells in the CCK-8 assay. Therefore, the development of bi-layered chitosan-based microrobots offers a general solution for using magnetic microrobots in biomedical applications by providing an easy-to-fabricate, highly mobile microrobotic platform with the incorporation of biocompatible natural polymers for enhanced biocompatibility.

## 1. Introduction

Micro/nanorobot-assisted interventional technologies have enabled minimally invasive medical operations in many deep parts of the body that are inaccessible through conventional means [1,2,3]. As such, micro/nanorobot is specially studied in the biomedical field as it has great potential in minimally invasive surgeries; thus, reducing surgical incisions, minimizing pain, and shortening recovery time [4,5,6]. When combined with magnetic control, micro/nanorobots can be remotely controlled to precisely target diseased areas in various environments, such as the nervous system, the fetus, and the circulatory system [7]. However, many previously reported micro/nanorobots consisted of non-biocompatible materials; this is because the fabrication methods had limitations on the type of material that could be used [8,9,10]. For example, a common material used for microrobots is SU-8 photoresist, which is not completely biocompatible and may lead to biofouling [11]. Moreover, magnetic microrobots were commonly coated with a nanolayer of nickel, which is not biocompatible and may cause adverse health effects, including cardiovascular, kidney, and lung diseases [12]. Therefore, developing new materials or strategies to easily incorporate biocompatible material is an important step to promote the development of biomedical micro/nanorobots [10,13]. Several previous studies tried to address this issue by using biocompatible materials or coating a layer of biocompatible material on micro/nanorobots [14,15]. For example, Wu et al. fabricated nanorobots with a biocompatible coating of chitosan and alginate for drug delivery [16]. These studies have shown that selecting the appropriate material, such as natural biocompatible materials, to fabricate biomedical micro/nanorobot is one of the most promising approaches.

Chitosan is the second most plentiful natural polysaccharide derived from chitin and processes delicate properties, including biocompatibility, biodegradability, sustainability, nontoxicity, absorption properties, and so on [17,18,19]. Chitosan has been widely utilized in a variety of areas, such as biomedical applications, the chemical industry, special or advanced materials, the food industry, and the environmental protection field [20,21]. There have already been reports on a few micro/nanorobots being made from chitosan or coated with chitosan to reduce cytotoxicity for medical applications [22]. For instance, Go et al. [23] reported magnetic microrobots tailored from chitosan microscaffolds by laser micromachining; these microrobots showed good biocompatibility and were used for liver cancer therapy and knee cartilage regeneration. Chen et al. [24] utilized magnetic chitosan microspheres as the inner layer and calcium alginate hydrogel as the outer layer to make a double-layer microrobot; these microrobots were nontoxic to cells and could be used for drug delivery. However, these reported microrobots that incorporated chitosan required complicated fabrication processes, needed expensive equipment, or used chitosan as an additive component [2,22,23,24,25,26]. There are few reports on using chitosan as the mainframe material to fabricate microrobots at the microscale and apply them in the biomedical area.

The actuation methods of microrobots are classified into physical methods (e.g., magnetic field, electric field, and acoustic wave), chemical methods (e.g., catalytic reactions), and biological methods (e.g., micro-organism-powered) [27,28,29,30]. In particular, magnetic actuation has the advantages of deep penetration, and remote control, making it ideal for manipulating micro/nanorobots at low Reynolds numbers [31,32,33]. Additionally, the motion of microrobots actuated by rotating magnetic fields (RMFs) can be precisely controlled by changing the field’s frequency, intensity, and direction [34,35]. Microrobots can be made responsive to magnetic fields by incorporating magnetic nanoparticles within their bodies [8,36]. The majority of magnetic microrobots are mainly fabricated through top-down techniques, such as 3D direct laser writing and glancing angle deposition, or bottom-up techniques, such as chemical synthesis and self-assembly [37,38,39,40,41,42]. However, these methods are either high cost and low throughput or very limited in the type of shapes that can be produced, demonstrating poor versatility. In comparison, photolithography is a common technique that is widely used in the semiconductor industry and large-scale mass production, especially the planar patterns [43]; thus, achiral planar microrobots fabricated using photolithography can overcome the aforementioned disadvantages. However, previously reported magnetic planar microrobots that were fabricated this way have some drawbacks. For example, the magnetic planar microrobots reported by Chen et al. were coated with a nickel layer, which is not a biocompatible material [44].

In this work, we developed magnetic achiral planar microrobots that were mass-fabricated through a lithography-based layer-by-layer strategy. The layer-by-layer strategy allowed for the fabrication of bi-layered achiral planar microrobots consisting of a top mainframe layer with chitosan and a bottom magnetic layer with biocompatible Fe_3_O_4_ nanoparticles. Similar to previously reported achiral planar microrobots, these bi-layered microrobots can convert rotational motion into translational motion to swim at a low Reynolds number when they are actuated by an RMF and can be precisely controlled. Furthermore, the lack of toxicity on the L929 cells makes the microrobots a promising candidate for future biological applications. Therefore, this work demonstrated the high-throughput fabrication of biocompatible magnetic microrobots in a very simple way by using biocompatible chitosan, which can serve as a platform for the future development of functional biomedical microrobots for applications such as targeted cell delivery.

## 2. Materials and Methods

### 2.1. Materials

Chitosan (CAS: 9012-76-4) with a viscosity of 100–200 mPas and a deacetylation degree of ≥95% was obtained from Shanghai Aladdin Biochemical Technology Co., Ltd. (Shanghai, China). Ferroferric oxide (Fe_3_O_4_) nanoparticles (CAS: 1317-61-9) with a diameter of 20 nm were purchased from Shanghai Macklin Biochemical Co., Ltd. (Shanghai, China). *N*,*N*′-methylenebis(acrylamide) (MBAA) (CAS:110-26-9) was obtained from Aladdin Industrial Corporation (Shanghai, China). Acrylamide (CAS:79-06-1) was obtained from Sun Chemical Technology (Shanghai) Co., Ltd. (Shanghai, China). Acetic acid (CAS: 64-19-7) was provided by Shanghai Aladdin Biochemical Technology Co. Dulbecco’s modified Eagle’s medium (DMEM) was supplied by Biological Industries (Kibbutz Beit-Haemek, Israel). Cell Counting Kit-8 (CCK-8) was obtained from MedChemExpress LLC. (Monmouth Junction, NJ, USA). Anticoagulant sheep blood was provided by Guangzhou Hongquan Biotechnology Co., Ltd. (Guangzhou, China). Mice Serum was provided by Guangzhou Dingguo Biotechnology Co., Ltd. (Guangzhou, China). Catalase and Protease K were provided by Shanghai Bepharm Science &Technology Co., Ltd. (Shanghai, China). Trypsin enzyme was obtained from Gibco (Grand Island, NY, USA). Other chemical reagents of analytical grade were supplied by Shanghai Sinopharm Chemical Reagent Co., Ltd. (Shanghai, China). Distilled water was used in the experiments.

### 2.2. Fabrication of Chitosan-Based Microrobots

#### 2.2.1. Preparation of Photo-Crosslinkable Chitosan Solution

Photo-crosslinkable chitosan solution (PC-CS, 6 wt%) was made by dissolving 1.2 g chitosan powder in 18.8 g of an aqueous solution consisting of 2 wt% of acetic acid at room temperature under mechanical stirring until a transparent and yellowish viscous solution was obtained. Then, 2 g acrylamide and 0.44 g MBAA were added and stirred, followed by dropping 3.2 mL K_2_S_2_O_8_ aqueous solution with a concentration of 0.05 mg/mL to form the PC-CS. The obtained solution was stored at a 4 °C refrigerator before use.

#### 2.2.2. Preparation of Magnetic Solution

Chitosan powder (CS, 0.15 g) and Fe_3_O_4_ nanoparticles (1.5 g) with a diameter of 20 nm were dissolved in 5 g of acetic acid (2 wt%) at room temperature using mechanical stirring until a black viscous solution was obtained. The magnetic solution (MS) was stored at 4 °C in a refrigerator.

#### 2.2.3. Design of Microrobots

The structure of the achiral planar microrobots was designed in AutoCAD and shown in Figure S1. The structure parameters were optimized according to previous work [44,45,46,47]. As shown in Figure S1, the width (W) of the microrobots was 100 μm, the length (L) was 200 μm, and the angle (θ) was 120°. The thickness of the microrobots was measured after the fabrication. The CAD drawing was used to create an array of 1400 microrobots on the photomask.

#### 2.2.4. Fabrication Procedure for the Microrobots

**Figure 1:**
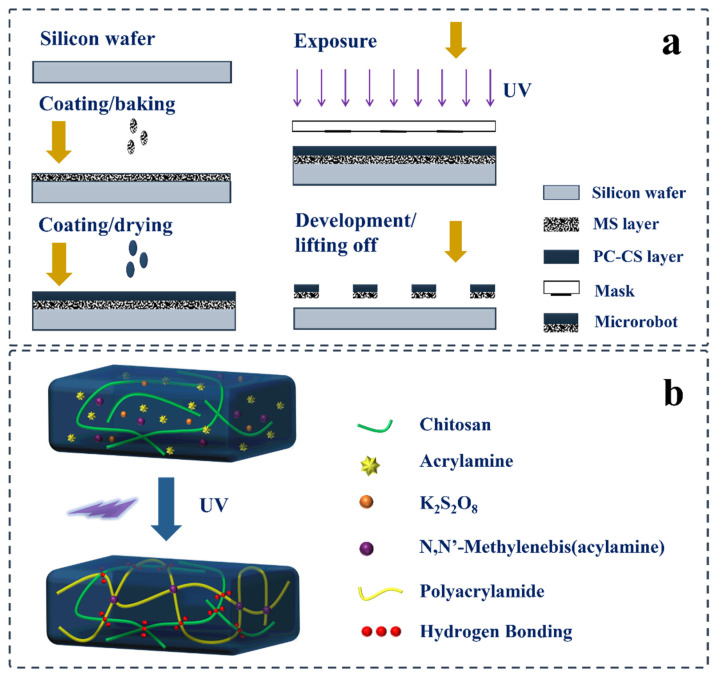
(**a**) Fabrication of chitosan-based achiral planar microrobots. (**b**) Schematic illustration of the photo-crosslinking process for the top layer.

Microrobots were fabricated using a layer-by-layer technique, as shown in Figure 1. The MS was used to form the bottom magnetic layer, while the PC-CS was used to construct the mainframe of the microrobots as the top layer. The bottom layer provided magnetism for magnetic actuation, and the top layer ensured biocompatibility and the mainframe of the microrobot. The fabrication process consisted of the following steps, as shown in Figure 1a: (1) cleaning the silicon wafer using a plasma cleaner (HARRICK PLASMA, PDC-002-HP, Ithaca, NY, USA); (2) spin-coating (1500 rpm, 60 s) and baking (65 °C, 10 min) the MS to form the magnetic film on the wafer; (3) spin-coating (3000 rpm, 60 s) and drying the PC-CS at ambient temperature on the magnetic film to form the photo-crosslinkable chitosan film; (4) exposing the sample under UV light (17 ± 0.2 mW/cm^2^) to transfer the achiral planar patterns from the photomask to the photo-crosslinkable chitosan film; (5) developing and lifting off the microrobots in 2 wt% acetic acid; (6) washing the microrobots with distilled water. These acquired microrobots were subsequently characterized.

### 2.3. Characterization

**Figure 2:**
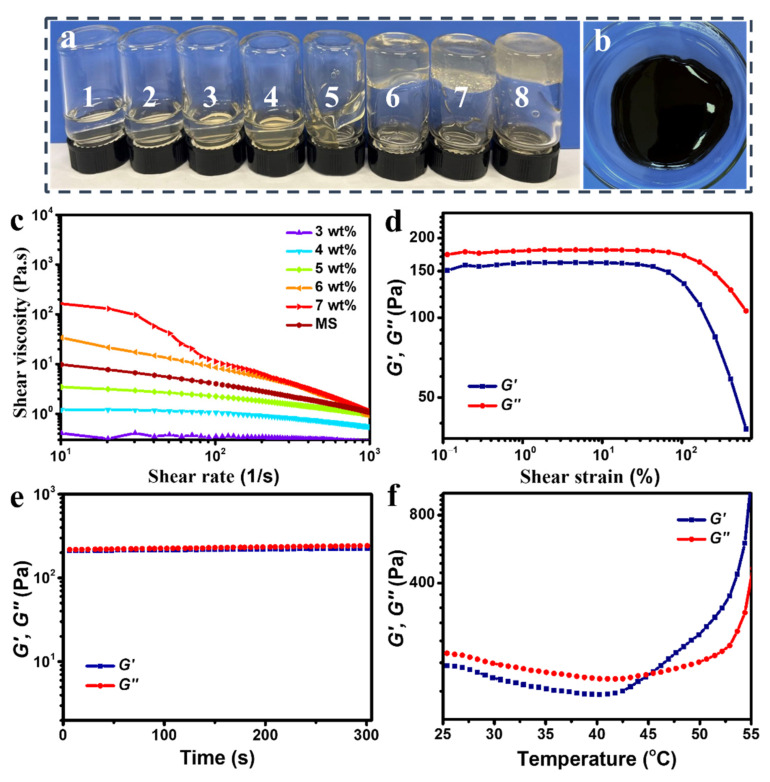
(**a**) Photograph of PC-CS at different concentrations (labels 1 to 8 refer to the concentration of 1 wt% to 8 wt%. (**b**) Photograph of an MS (3 wt%). (**c**) The apparent viscosity of PC-CS at different concentrations plotted as a function of shear rate. (**d**) Shear and loss storage modulus of PC-CS (6 wt%) with strain varying from 0.1% to 700% at a fixed frequency of 1 Hz. (**e**) Shear and loss storage modulus of PC-CS (6 wt%) with time changing from 0 to 300 s. (**f**) Shear and loss storage modulus of PC-CS (6 wt%) with temperature increasing from 25 °C to 55 °C.

Photographs of the PC-CSs and MS are shown in Figure 2a,b. The rheological behavior of the PC-CSs was tested using a rotational rheometer (HAAKE MARS III, Thermo Fisher Scientific, Cambridge, UK) with 20 mm parallel plates at a gap of 1.0 mm. The apparent shear viscosity was monitored by increasing the shear rate from 10^1^ to 10^3^ s^−1^. Oscillatory measurements were measured at a constant frequency of 1 Hz and increasing strain from 10^−1^ to 7 × 10^2^%. All the measurements were carried out at 25 °C. Oscillatory time sweeps were performed in 300 s at 10% strain with a frequency of 1 Hz.

The infrared transmittance was measured using a Fourier transform infrared (FTIR) spectrometer (SHIMADZU, IRAffinity-1S, Kyoto, Japan) with the wavenumber from 400–4000 cm^−1^ at ambient temperature. X-ray diffraction (XRD) measurements were carried out using an X-ray diffractometer (Rigaku Smartlab, Tokyo, Japan) with Cu Kα radiation (λ = 1.54 Å) at 40 kV, 30 mA. A vibrating sample magnetometer (VSM, LakeShore 7404, Westerville, OH, USA) was used to evaluate the magnetic characteristics of Fe_3_O_4_ nanoparticles and microrobots.

The morphology of microrobots was observed using microscopy (Mshot, MS23-H, Guangzhou, China). The surface morphology and the elemental composition of the microrobots were characterized using scanning electron microscopy (SEM, ZEISS, Merlin, Oberkochen, Germany) at 5 keV and 1 nA and energy-dispersive spectrometry (EDS, EDAX, Mahwah, NJ, USA, Octane Pro), respectively. The structural stability of microrobots in different media was characterized by observing the microrobots’ morphology through a microscope (Mshot, MS23-H, Guangzhou, China).

### 2.4. Motion Test of the Microrobots

A three-dimensional Helmholtz coil system consisting of three pairs of orthogonal coils was used to generate RMFs to actuate the microrobots for the motion control experiments. The microrobots were placed in distilled water in a glass dish at the center of the Helmholtz coil system to ensure the uniformity of the rotating magnetic field. An optical microscope with a 2× objective lens and a CMOS camera (PCO-TECH Inc., Lower Bavaria, Germany) was used to observe the motion of microrobots. To evaluate the motion of the microrobots, recorded videos were analyzed using a MATLAB tracking algorithm and the *x* and *y* positions of microrobots were used to calculate the average movement speed.

### 2.5. In Vitro Cytotoxicity

Cell viability was evaluated by employing the CCK-8 assay. Mouse mammary carcinoma L929 cells were used to test the cytotoxicity of microrobot material. The cells (4 × 10^5^) were cultured in a Petri dish in DMEM supplemented with 10 vol% fetal bovine serum and 1 vol% penicillin/streptomycin at 37 °C in a CO_2_ atmosphere (5 vol%). After 24 h, crushed and sterilized microrobots were added in with the final concentrations of 0.2, 0.5, 0.8, 1.0, and 2.0 mg/mL, respectively, and cultured for 1, 3, and 7 days with culture media exchanged every 48 h. CCK-8 (Dojindo, Kumamoto, Japan) was used to determine the survival of the cells. The absorbance at 450 nm was recorded 2 h later using a multifunctional full wavelength microplate reader (INFINITE 200 PRO, Tecan Austria GmbH, Grödig, Austria/Europe). Triplicate experiments were performed each time, and cells with PBS were cultured as the control experiment. A live/dead staining kit (CalceinAM/PI, Biyuntian, Tonghua, China) was used to distinguish living from dead cells under a fluorescence microscope (Nikon, Tokyo, Japan) after the sterilized microrobots were co-cultured for 24 h.

## 3. Results

### 3.1. Fabrication of the Microrobots

In this study, the microrobots with the structure of an achiral planar design were patterned through photolithography. This was possible due to the polymerization of acrylamide in the PC-CS; the crosslinking process between the polymerized acrylamide and chitosan by the crosslinker of MBAA and hydrogen bond is shown in Figure 1b. After performing the UV exposure, the UV photolysis K_2_S_2_O_8_ and water generated the free radicals of OH· and SO_4_^−^· triggered the polymerization of acrylamide and further propagated the chain reaction. In rapid sequence, the crosslinking between the polyacrylamide (PAAm) chain and chitosan occurred to obtain the network with dimensional stability for the achiral planar shape of the microrobots.

In order to prepare the appropriate PC-CS, which could not only crosslink to form the stable structures but also form the specific achiral planar shapes with clear edges, the effects of the concentration of the PC-CS on the fabrication of microrobot were systematically investigated. Labels 1–8 from Figure 2a show PC-CSs (dissolved in 2 wt% acetic acid) with the concentration increasing from 1 wt% to 8 wt%. The corresponding apparent shear viscosities of the PC-CSs increased with increasing concentration, as shown in Figure 2c. The apparent viscosity at a shear rate of 10 s^−1^ increased from 0.41 Pa.s to 169.74 Pa.s as the concentration increased from 3 wt% to 7 wt%. The high viscosity at higher concentrations of chitosan indicated stronger entanglement of chitosan chains within the solution. For concentrations less than or equal to 5 wt%, the solution showed good fluidity but could not form stable structures due to insufficient chitosan chains (Figure 2a labels 1–5). For concentrations greater than or equal to 7 wt%, the viscosity of the solution was too high to be used for photo-crosslinking due to the physically gel-like behavior of the solution (Figure 2a 7–8). The solution with the concentration of 6 wt% had an appropriate viscosity and can be crosslinked into stable structures; thus, the 6 wt% PC-CS was used to fabricate the microrobots.

From the rheological measurements, the PC-CS clearly demonstrated typical shear-thinning (Figure 2c PC-CS) and shear-yielding behaviors (Figure 2d). The apparent viscosity of PC-CS (6 wt%) decreased sharply from 34.00 Pa.s to 0.96 Pa.s, with the shear rate increasing from 10^1^ s^−1^ to 10^3^ s^−1^. While the shear thinning behavior has an effect on the thickness of the film from spin coating, it is still possible to control the film thickness [48]. To investigate the viscoelastic properties of the PC-CS, oscillatory measurements were carried out to measure the storage modulus (*G′*) and loss modulus (*G″*). Figure 2d shows that the loss modulus (*G″*) (≈180 Pa) of PC-CS was higher than the storage modulus (*G′*) (≈160 Ps) in the linear viscoelastic region with the range of stain from 0.1% to 43%, indicating the PC-CS will have the fluidity needed to be spin-coated into thin films under a wide range of strain. Moreover, Figure 2e shows the storage modulus (*G′*) and loss modulus (*G″*) could keep at a constant value with time changing from 0 to 300 s, indicating that the PC-CS is stable in viscoelasticity for 300 s; this will benefit the spin-coating process to form a uniform thickness of the film. Figure 2f shows the storage modulus (*G′*) was higher than the loss modulus (*G″*) after 45 °C, indicating that the fluidity gradually weakened with the temperature continuously increasing. Thus, it will be appropriate to spin coat PC-CS at ambient temperature and dry it at a temperature below 45 °C.

The MS, which contained CS (3 wt%) with Fe_3_O_4_ nanoparticles dispersed evenly to be well spin-coated on the silicon wafer, is shown in a photograph in Figure 2b. The apparent viscosity of the MS is shown in Figure 2c. Compared to the PC-CS at 3 wt%, the addition of Fe_3_O_4_ nanoparticles substantially enhanced the viscosity of the solution from 0.41 Pa.s to 9.81 Pa.s with the shear rate of 10^1^ s^−1^; this is a strong indication of the interaction between the chitosan polymer and the Fe_3_O_4_ nanoparticles.

### 3.2. Structure of the Microrobots

**Figure 3:**
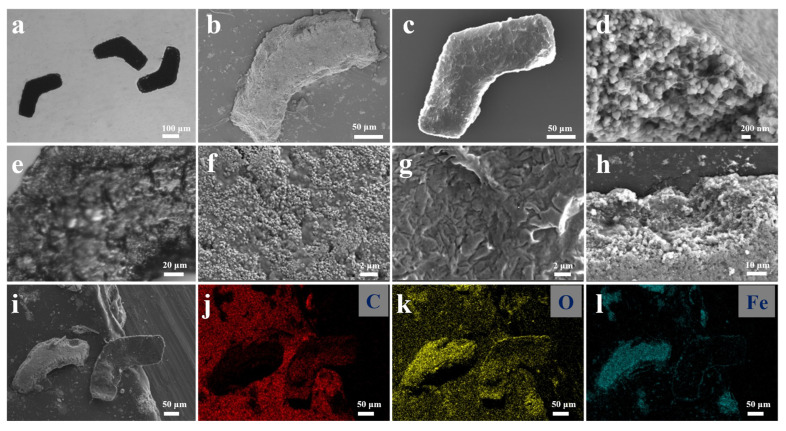
Optical images with (**a**) 5× and (**e**) 40× objective lens of chitosan-based microrobots. SEM (**b**) and zoomed-in (**f**) images of a microrobot with the bottom layer faced up. SEM (**c**) and zoomed-in (**g**) images of a microrobot with the top layer faced up. (**d**) Zoomed-in SEM image of the bottom layer showing nanoparticles embedded in the magnetic layer. (**h**) SEM image of the tilted cross-section of a microrobot. (**i**–**l**) EDAX mapping of two microrobots.

The morphology and element mapping of the microrobots are shown in Figure 3. The microrobots in water appeared black because of the magnetic layer, which contained Fe_3_O_4_ nanoparticles, as shown in Figure 3a. The shapes of the microrobots visually matched the achiral planar design from the CAD drawing, indicating that patterns on the photomask were successfully transferred to the chitosan bi-layer to create the microrobots. The bottom surface of a representative microrobot with the magnetic layer appeared rough, as shown in the optical image in Figure 3e and the SEM image in Figure 3b. A zoomed-in SEM image in Figure 3f further verifies the rough surface morphology of the magnetic layer. The rough surface was due to the Fe_3_O_4_ nanoparticles and soft chitosan [19]. The top surface created by the PC-CS without Fe_3_O_4_ nanoparticles was also rough, as shown with a representative microrobot in the SEM images in Figure 3c and the zoomed-in SEM image in Figure 3g. In comparison, the zoomed-in SEM images revealed that the surface morphology of the bottom (Figure 3f) and top (Figure 3g) surfaces are very different due to the concentration of the chitosan and the presence of Fe_3_O_4_ nanoparticles. To examine the inside of the magnetic layer, SEM images were taken of an upside-down microrobot with a damaged area that revealed the inside of the magnetic layer. The Fe_3_O_4_ nanoparticles were clearly embedded within the chitosan network of the magnetic layer, as shown in Figure 3d. In conjunction with the SEM image from Figure 3f, where the nanoparticles were attached to the surface of the layer, it can be seen that the Fe_3_O_4_ nanoparticles were uniformly distributed and adhered throughout the chitosan network. To examine the adhesion between the two layers (magnetic and PC-CS layers), an SEM image of a tilted microrobot showing the interface between the two layers is shown in Figure 3h; it can be seen that the two layers are in full contact, indicating that the two layers had adhered together. EDAX analysis (Figure S2) of the Fe element map further confirmed the bi-layer structure of the microrobots by showing a layer with Fe and a layer without Fe. The EDAX element maps of microrobots with the top layer and the bottom layer faced up (located left and right, respectively, in Figure 3i) further verified that the microrobots were constructed with two layers, as visualized by the density of colored pixels for C, O, and Fe elements in Figure 3j–l. The microrobot with the bottom layer faced up showed fewer pixels for the C element map (Figure 3j) and showed more pixels (Figure 3l) for the Fe element map, revealing the different layers of the microrobots. The Fe element map of the bottom layer further verified the Fe_3_O_4_ nanoparticles were evenly dispersed (Figure 3l). As the two layers both had the O element, the O element map shows minor differences in pixels (Figure 3k). Figure S3 shows the images of microrobots after submerging them in anticoagulant sheep blood and mice serum at 37 °C with a rotating speed of 100 rpm for 24 h. This indicated that the microrobots were about to maintain their L-shaped structure after the test, showing that the microrobots have good structural stability. Additionally, when placed in catalase (20 U/mL), protease K (0.2 mg/mL), and trypsin enzyme (0.25%), the microrobots were able to maintain their L-shaped structure at 37 °C for 24 h, further verifying their structural stability.

**Figure 4:**
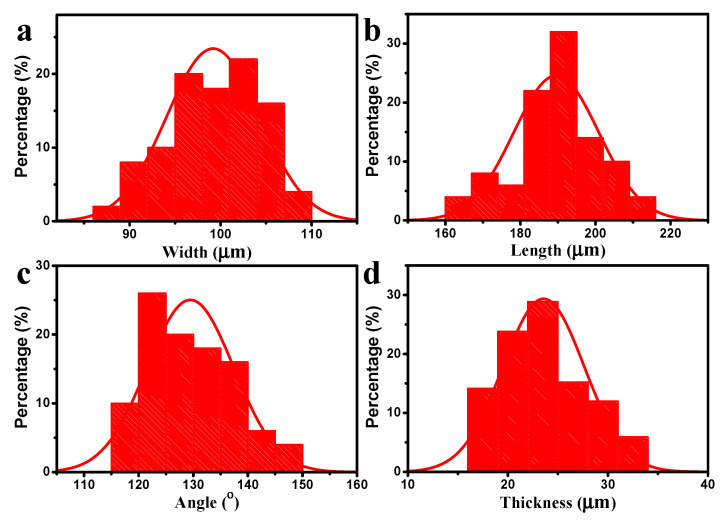
The (**a**) width, (**b**) length, and (**c**) included angle distributions of chitosan-based microrobots in water. (**d**) Thickness distribution of microrobots in the dry state.

The size distribution of the chitosan-based microrobots is shown in Figure 4. As shown in Figure 4a–c, the width, length, and included angle distributions yielded an average of 99.18 ± 5.11 μm, 189.56 ± 11.37 μm, and 129.46 ± 7.97°, which were close to the pre-designed size parameters of 100 μm, 200 μm, and 120° with a slight deviation, indicating that this layer-by-layer method performed well to fabricate the chitosan-based achiral microrobots. Figure 4d shows the thickness distribution of microrobots yielded an average thickness of 23.56 ± 4.08 μm in the dry state. For comparison, the thickness distribution of the structures obtained from photo-crosslinking the PC-CS (without magnetic layer) under the same fabrication conditions yielded an average thickness of 8.25 ± 3.35 μm, as shown in Figure S4.

### 3.3. Physiochemical Property of the Microrobot

**Figure 5:**
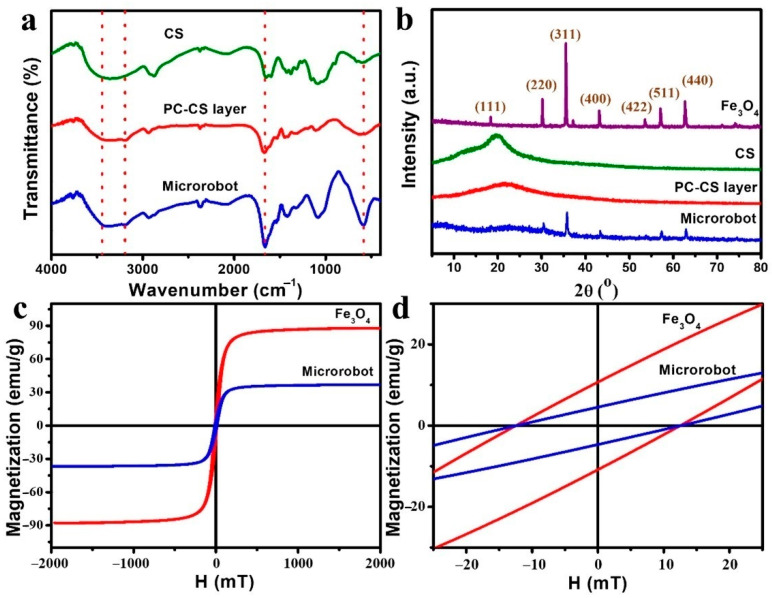
(**a**) FTIR spectra of pure chitosan, the top layer of the microrobots, and the microrobots. (**b**) XRD patterns of Fe_3_O_4_ nanoparticles, chitosan, the top layer of the microrobots, and the microrobots. (**c**) Magnetic hysteresis loops of Fe_3_O_4_ nanoparticles and the microrobots. (**d**) Zoomed-in view where the hysteresis loops of the Fe_3_O_4_ nanoparticles and the microrobots intersect.

The FTIR spectra of pure chitosan, the top layer of the microrobots, and the microrobots are shown in Figure 5. The band at 3444 cm^−1^ was attributed to the stretching vibrations of -OH of chitosan. The new peaks at 3196 cm^−1^ for the stretching vibration of N-H, and 1654 cm^−1^ for C=O stretching of PAAm were from the top layer of the microrobots and the microrobots in comparison with pure chitosan. The characterized peak at 585 cm^−1^ was the absorption peak of Fe_3_O_4_. The results verified that the microrobots mainly consisted of chitosan, polymerized acrylamide, and Fe_3_O_4_ nanoparticles. The XRD spectra of Fe_3_O_4_, chitosan, the top layer of the microrobot, and the microrobot are shown in Figure 5b. Aside from the diffraction peak of chitosan at 2θ of 21.8° of chitosan and the top layer of the microrobot, the microrobots presented strong characteristic diffraction peaks for Fe_3_O_4_ at (111), (220), (311), (400), (422), (511), and (440), indicating the existence of Fe_3_O_4_ nanoparticles in the microrobots. The hysteresis loops for the Fe_3_O_4_ nanoparticles and the microrobots are shown in Figure 5c. The saturation magnetization of the Fe_3_O_4_ nanoparticles was 87.8 emu/g, showing a slightly lower value than the theoretical value of 92 emu/g. This was probably caused by agglomerations of the Fe_3_O_4_ nanoparticles [49]. The saturation magnetization of the microrobots was 37.0 emu/g, showing a much lower value than the pure Fe_3_O_4_ nanoparticles due to the existence of PC-CS. The Fe_3_O_4_ nanoparticles and the microrobots had the same coercivity of 12.58 mT since the Fe_3_O_4_ nanoparticles in both groups were the same (Figure 5d).

All these results further verified the successful fabrication of magnetic microrobots with the composition of chitosan, PAAm, and Fe_3_O_4_ nanoparticles.

### 3.4. Motion of Microrobots

To investigate the magnetically actuated mobility of the microrobots, a uniform RMF was generated by a 3D Helmholtz coil system (Figure S5). The motion of the microrobots was investigated under different RMF frequencies and intensities. The average velocity was obtained by using a tracking algorithm written in MATLAB.

**Figure 6:**
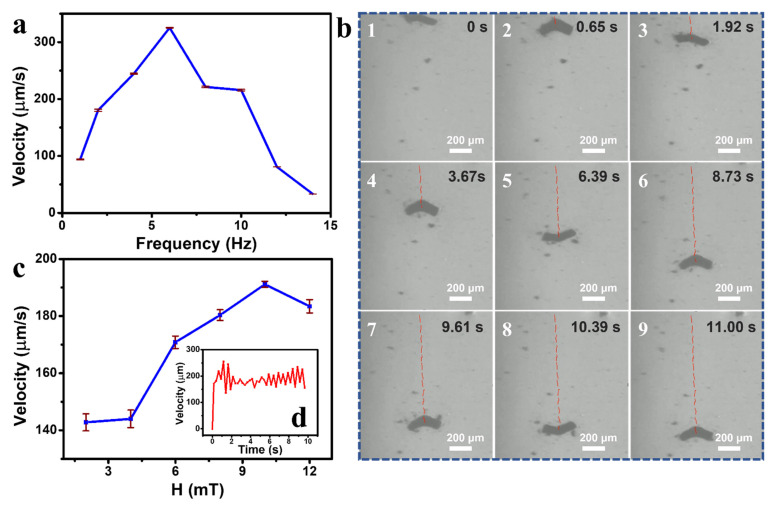
(**a**) Velocity profile of the chitosan-based microrobots from 1 to 14 Hz. (**b**) Positions of a representative microrobot moving at 1 Hz for 11 s. (**c**) Velocity profile of the microrobots with the RMF intensity increasing from 2 to 12 mT at 2 Hz. (**d**) The inset figure shows the velocities of the microrobot at 12 mT for 10 s.

In the first sets of experiments, microrobots were actuated using 8 mT RMFs ranging from 1–14 Hz. The average velocity of the microrobots increased from 93.84 μm/s at 1 Hz to 325.30 μm/s at 6 Hz, as shown in Figure 6a. After reaching the step-out frequency of 6 Hz, the velocity rapidly declined to 33.20 μm/s at 14 Hz; this is the typical step-out behavior for every previously reported magnetic microrobot [50]. Snapshots of a representative microrobot moving under the actuation of an 8 mT RMF at 1 Hz are shown in Figure 6b (please also see Video S1). The microrobot was able to move in a straight line, indicating that the microrobot can move steadily in water for 11 s under an RMF.

The average velocities of microrobots under a 2 Hz RMF with different magnetic field intensities (2 to 12 mT) are shown in Figure 6c. The average velocity of microrobots increased from 142.81 μm/s at 2 mT to 191.14 μm/s at 10 mT, and then slightly decreased to 183.42 μm/s at 12 mT. This is because the precession angle of the microrobot changed as the magnetic intensity increased; the same behavior was observed from previously reported achiral microswimmers [51]. The velocities of the microrobots with the intensity of 12 mT are shown for 10 s in the inset plot from Figure 6d. The velocities showed slight fluctuations during the observation period, demonstrating a steady motion of the microrobot.

The results from the motion control experiments indicated that the microrobots could be magnetically actuated to move steadily in a fluidic environment and controlled to move at different velocities by varying the frequency and intensity of the RMF, demonstrating the potential for targeted therapy.

### 3.5. Biocompatibility of Microrobot

**Figure 7:**
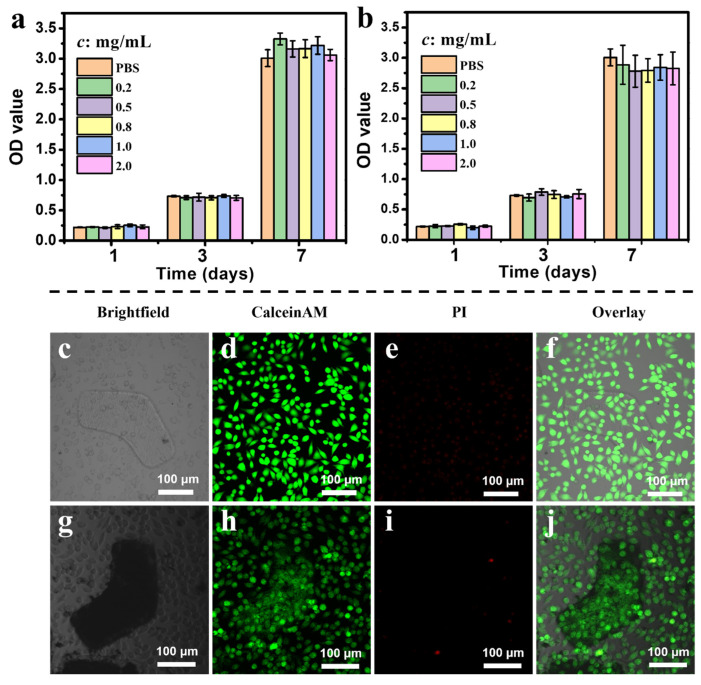
L929 viability of (**a**) smashed top layer material and (**b**) smashed microrobot material after 1, 3, and 7 days of incubation (Error bars represent standard deviations. *c* refers to concentrations of material dispersed in PBS). Fluorescence microscopy images of L929 cells stained by CalceinAM/PI dyes after co-cultured with the material from the top layer (**c**–**f**) and microrobots (**g**–**j**).

To validate the potential to use chitosan-based microrobots for biomedical applications, their biocompatibility with L929 cells was investigated via live/dead staining and CCK-8 assay (Figure 7). L929 cells were co-cultured with smashed materials at different concentrations in a culture medium, as shown in Figure 7a. The experiments compared six groups: PBS (control), 0.2 mg/mL, 0.5 mg/mL, 0.8 mg/mL, 1.0 mg/mL, and 2.0 mg/mL groups. These series of experiments were performed using the smashed top layer material from the top layer of the microrobots, which was created from the PC-CS without the magnetic layer, and the smashed microrobot material from the entire microrobots, which included both the top PC-CS layer and the bottom magnetic layer. According to the quantitative statistical analysis for 1, 3, and 7 days, there was an obvious increase in the OD values on days 3 and 7 (Figure 7b), indicating the excellent biocompatibility of the materials of PC-CS layer and the material of the microrobots. Fluorescent microscope images of the L-shaped PC-CS layer and the microrobots cultured with L929 cells are shown in Figure 7c–j, respectively. There were only a few dead cells after being co-cultured for 24 h, further verifying the excellent biocompatibility of the microrobots. These results indicated that these microrobots have great potential to be applied in the biomedical field due to the excellent biocompatibility of the material used for fabrication.

## 4. Discussion

Magnetically actuated microrobots have great potential in various fields, especially the biomedical field. While many previously reported magnetic microrobots consisted of non-biocompatible materials due to the limitations of the fabrication methods, this work tried to improve their biocompatibility by incorporating chitosan, which is a natural polymer. Through photolithography, a bilayer magnetic microrobot with the achiral planar design based on chitosan can be made using a layer-by-layer strategy. This sample and easy-operation fabrication method and strategy can be developed to fabricate planar or planar-based microrobots, microstructures, and microdevices based on other natural or synthesized polymers. Though there are some limitations in this work, such as the softness of the microrobot will cause minimal quivers while magnetic actuating, it offers a general platform to fabricate magnetic microrobots in a very simple way. Additionally, the microrobots might not be completely nontoxicity to the human body, as some formulations of photo-crossable chitosan were reported to cause negative side effects; this means in-depth studies will be needed to fully assess the safety of the material. Based on this work, future work will focus on developing more appropriate materials to construct fully nontoxic achiral planar microrobots, improving their surface properties and possibility applying them in the biomedical field. In the long-term run, magnetic microrobots are expected to be used in the biomedical field to perform sophisticated work under complicated environments such as invasive surgery, drug delivery, and treatment.

## 5. Conclusions

In summary, we have developed a versatile and simple approach to massively fabricate chitosan-based achiral planar microrobots by using photolithography combined with a layer-by-layer strategy. This fabrication approach can cost-efficiently mass-fabricate planar microrobots in parallel, which is significantly higher throughput compared to the fabrication process of 3D microrobots. The microrobots fabricated using this approach are more geometrically consistent than those fabricated by chemical synthesis or self-assembly. By employing the biopolymer of chitosan as the mainframe component, the obtained bi-layered microrobots consist of a bottom magnetic layer and a top photo-crosslinked chitosan layer. The structural and material characterization results verified that the microrobots were fabricated with dimensions close to the pre-designed structures on the photomask and consisted of elements corresponding to chitosan and Fe_3_O_4_ nanoparticles. Motion control test showed that the velocity of the microrobots could be tuned by changing the frequency or intensity of the rotating magnetic field. Finally, cell viability tests showed that the microrobots have excellent biocompatibility, which is expected since the microrobots were created entirely out of biocompatible materials; this strongly indicated that the microrobots could be a good candidate in biomedical applications at the microscale. This work will serve to provide a simple and practical approach to fabricating achiral microrobotic platforms to create biocompatible microrobots for potential applications in the biomedical field.

## Data Availability

Not applicable.

## Supplementary Materials

The following supporting information can be downloaded at: https://www.mdpi.com/article/10.3390/polym14245509/s1, Figure S1: Structure of the L-shaped microrobot; Figure S2: EDAX mapping of the tilted cross-section of a microrobot; Figure S3: Optical images with 5× objective lens of microrobots after immersing in anticoagulant sheep blood (a) and mice serum (b) at 37 °C with rotating speed of 100 rpm for 24 h. Figure S4: Thickness distribution of the top layer of microrobots in the dry state; Figure S5: A 3D Helmholtz coil system; Video S1: Motion of microrobot with the magnetic field of 8 mT and 1 Hz.
