# Supplementary material for: Fabrication of Bilayer Magnetically Actuated L-Shaped Microrobot Based on Chitosan via Photolithography
Source: Polymers (Basel). 2022 Dec 15;14(24):5509. doi: 10.3390/polym14245509 (PMC9784805; doi:10.3390/polym14245509)
Supplement: Supplementary file 1 [file polymers-14-05509-s001.zip › polymers-2044980-supplementary.pdf]

polymers

Supplementary Material

Fabrication of Bilayer Magnetically Actuated L-Shaped Microrobot Based on Chitosan via Photolithography

Haoying Wang [1,*], Xiaoxia Song [1], Junfeng Xiong [1] and U Kei Cheang [1,2,3,*]

1 Department of Mechanical and Energy Engineering, Southern University of Science and Technology, Shenzhen 518055, China
2 Shenzhen Key Laboratory of Biomimetic Robotics and Intelligent Systems, Southern University of Science and Technology, Shenzhen 518055, China
3 Guangdong Provincial Key Laboratory of Human-Augmentation and Rehabilitation Robotics in Universities, Southern University of Science and Technology, Shenzhen 518055, China
* Correspondence: wanghy3@sustech.edu.cn (H.W.); cheanguk@sustech.edu.cn (U.K.C.)

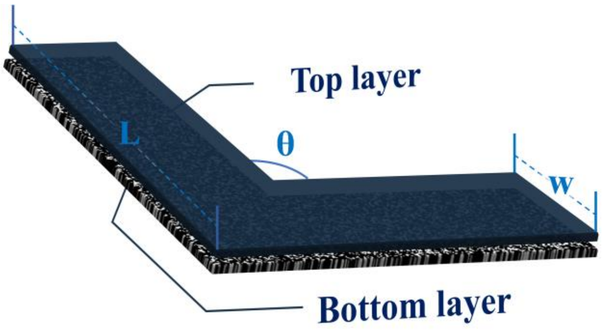

Figure S1. Structure of the L-shaped microrobot.

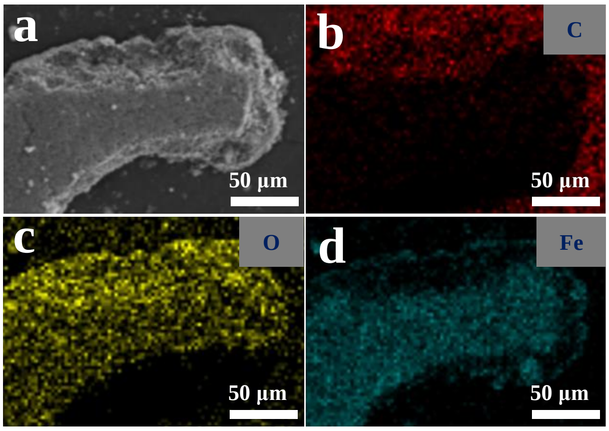

Figure S2. EDAX mapping of the tilted cross-section of a microrobot.

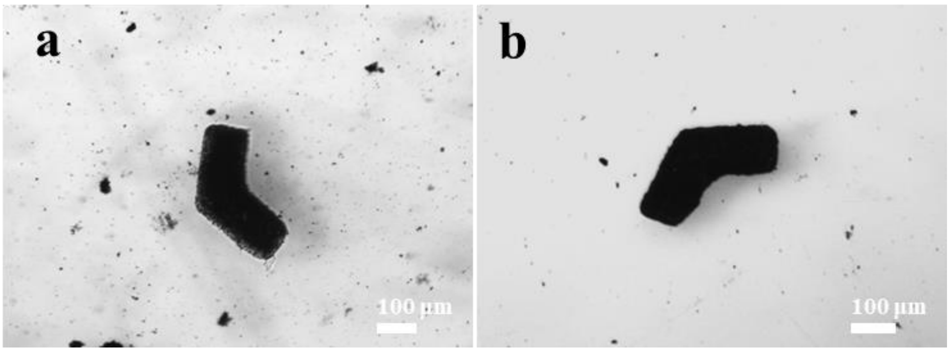

Figure S3. Optical images with 5× objective lens of microrobots after immersing in anticoagulant sheep blood (a) and mice serum (b) at 37 ℃ with rotating speed of 100 rpm for 24 h.

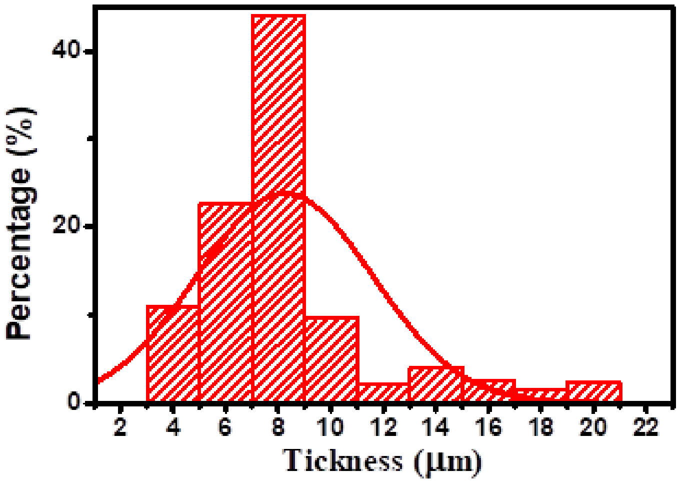

Figure S4. Thickness distribution of the top layer of microrobots in dry state.

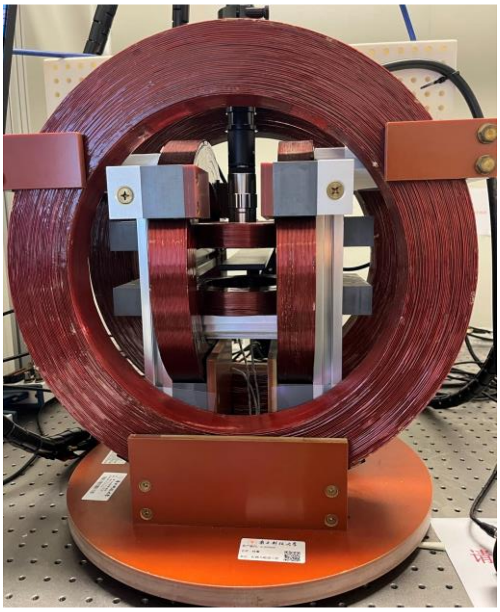

Figure S5. A 3D Helmholtz coil system.
